# Transcriptome-wide analyses of RNA m6A methylation in hexaploid wheat reveal its roles in mRNA translation regulation

**DOI:** 10.3389/fpls.2022.917335

**Published:** 2022-08-25

**Authors:** Tao Huang, Wei-Jie He, Cheng Li, Jing-Bo Zhang, Yu-Cai Liao, Bo Song, Peng Yang

**Affiliations:** ^1^Molecular Biotechnology Laboratory of Triticeae Crops, Huazhong Agricultural University, Wuhan, China; ^2^College of Life Science and Technology, Huazhong Agricultural University, Wuhan, China; ^3^College of Plant Science and Technology, Huazhong Agricultural University, Wuhan, China; ^4^College of Agriculture, Shihezi University, Shihezi, China; ^5^Shenzhen Branch, Guangdong Laboratory for Lingnan Modern Agriculture, Genome Analysis Laboratory of the Ministry of Agriculture, Agricultural Genomics Institute at Shenzhen, Chinese Academy of Agricultural Sciences, Shenzhen, China; ^6^Jiangsu Ruihua Agricultural Science and Technology Co., Ltd., Suqian, China

**Keywords:** m6A, N6-methyladenosine, RNA modification, small RNA, translation, wheat

## Abstract

N6-methyladenosine (m6A) is the most abundant RNA modification in eukaryotic messenger RNAs. m6A was discovered in wheat about 40 years ago; however, its potential roles in wheat remain unknown. In this study, we profiled m6As in spikelets transcriptome at the flowering stage of hexaploid wheat and found that m6As are evenly distributed across the A, B, and D subgenomes but their extents and locations vary across homeologous genes. m6As are enriched in homeologous genes with close expression levels and the m6A methylated genes are more conserved. The extent of m6A methylation is negatively correlated with mRNA expression levels and its presence on mRNAs has profound impacts on mRNA translation in a location-dependent manner. Specifically, m6As within coding sequences and 3′UTRs repress the translation of mRNAs while the m6As within 5′UTRs and start codons could promote it. The m6A-containing mRNAs are significantly enriched in processes and pathways of “translation” and “RNA transport,” suggesting the potential role of m6As in regulating the translation of genes involved in translation regulation. Our data also show a stronger translation inhibition by small RNAs (miRNA and phasiRNA) than by m6A methylation, and no synergistical effect between the two was observed. We propose a secondary amplification machinery of translation regulation triggered by the changes in m6A methylation status. Taken together, our results suggest translation regulation as a key role played by m6As in hexaploid wheat.

## Introduction

Post-transcriptional modifications of nucleotide bases on RNAs are prevalent and are particularly enriched in non-coding RNAs, such as tRNAs (Gilbert et al., 2016). Recent technical advances have enabled transcriptome-wide identification of various modified RNA bases including N6-methyladenosine (m6A), 5-methylcytidine (m5C), and pseudouridine (ψ) in diverse organisms including bacteria, yeasts, humans and other animals, and plants (Edelheit et al., 2013; Luo et al., 2014; Wan et al., 2015; Wang et al., 2017). Among them, m6A is the most abundant internal modification of mRNAs. The modification of mRNAs can have physiological consequences in post-transcriptional processes. For example, m6A methylation on pri-miRNA is required for the biogenesis of microRNAs (Alarcón et al., 2015). N6-methyladenosine methylation on mRNAs can regulate the translation of methylated mRNAs (Wang et al., 2015), reduce the stability of mRNA (Geula et al., 2015), switch the structure of mRNAs, and affect their interactions with proteins (Liu et al., 2015).

In recent years, many studies have enhanced our understanding of the roles of m6A methylation in plants. There are lines of evidence suggesting that the m6A methylation of mRNAs are widely engaged in various processes of plants, including embryo development (Zhong et al., 2008; Bodi et al., 2012), photosynthesis (Luo et al., 2014; Wan et al., 2015), callus induction (Li et al., 2014; Du et al., 2020), as well as responses to biotic or abiotic stresses (Bai et al., 2021; Cheng et al., 2021; Zhang et al., 2021; Yue et al., 2022). For example, the knock-out of a mRNA adenosine methylase (MTA) gene in *Arabidopsis* resulted in the inhibition of m6A methylation and the failure of embryo development (Zhong et al., 2008). N6-methyladenosine methylation of mRNAs are known to be involved in many molecular processes in mammals, such as the regulation of RNA splicing, export, and translation, miRNA processing and others (Gilbert et al., 2016) but its roles in plants are relatively poorly understood. Recent studies revealed some roles of m6A in plants similar to those observed in mammals, including the stabilization of mRNAs (Anderson et al., 2018), the prevention of chimeric mRNAs (Pontier et al., 2019), and the regulation of mRNA translation (Luo et al., 2020).

It is noteworthy that most of these studies were conducted in diploid organisms with simple genomes, such as *Arabidopsis*, rice, maize, or tomato; thus, more investigations are required to expand our understanding of the features of m6A methylation and its roles in polyploidy genomes. Bread wheat (*Triticum aestivum* L.) is one of the most important staple crops in the world. It has a hexaploidy genome (2n = 6× = 42, AABBDD) consisting of three sub-genomes, which is believed to be a result of two rounds of hybridization speciation, with the latest hybridization event occurring between a domesticated emmer (*T. dicoccum*, AABB) and a goatgrass (*Aegilops tauschii*, DD) ∼8500 years ago. Due to this short period of speciation of bread wheat, many homeologous genes across the sub-genomes are functionally redundant and mutual complementary (Peng et al., 2011; Levy and Feldman, 2022). Many genomic blocks including genes in sub-genomes can be well aligned. Ramírez-González et al. (2018) identified 18,474 homeologous gene triads, which consist of genes that have 1:1:1 correspondence across the A, B, and D sub-genomes, 17,400 of which were found within syntenic blocks across sub-genomes. These provide an opportunity for us to explore the variation and conservation of m6A methylation across sub-genomes, as well as the patterns of translation regulation in the background of a polyploid genome. To further our understanding of the features and functions of m6A in wheat, we performed transcriptome-wide profiling of m6A in wheat and found higher conservation of m6A methylated genes than those not methylated. We observed negative correlation between m6A methylation levels and mRNA abundance and translation efficiency. Specifically, m6A methylation within mRNA coding sequences and 3′UTR appears to represses the translation of mRNAs while methylation at the 5′UTR and start codon could act as a translation enhancer. Both m6A methylation and small RNA binding suppressed wheat mRNA translation but no synergism between them was found. Finally, we propose a model of secondary amplification of translational regulation by m6A, which might allow rapid response to stresses.

## Materials and methods

### Plant materials

To better investigate the regulation of translation by m6A methylation or small RNAs, spikes of the hexaploid wheat cultivar Chinese Spring (*T. aestivum* L.) at the flowering stage were used in this study. Briefly, the spikelets from the middle of spikes were spliced off and frozen immediately in liquid nitrogen for 5 min before they were transferred into fresh Eppendorf tubes. The frozen spikelets were maintained at −80°C until they were used for further experiments. Approximately twelve spikelets (∼0.8 g) were collected for each replicate.

### RNC-seq

The ribosome-nascent chain complex (RNC) was extracted as described by Esposito et al. (2010) and Wang et al. (2013) with modifications. Briefly, about 0.2 g of spikelets were ground and incubated in cycloheximide (100 μg/ml) for 15 min and then rinsed using phosphate buffered saline, followed by incubation in ice in lysis buffer (1% Triton X-100, 20 mM HEPES-KOH, 15 mM MgCl_2_, 200 mM KCl, 100 μg/ml cycloheximide, and 2 mM dithiothreitol) for 30 min. Debris was removed by centrifuging at 16,200 × *g* for 10 min at 4°C. RNC was collected from the resulting supernatants by ultra-centrifugation at 330,000 × *g* at 4°C for 3 h in 30% sucrose buffer. RNC-associated RNA and total RNA were then separately extracted using TRIzol and purified using oligo(dT)-attached magnetic beads and subjected to standard protocols for RNA-seq (Zhu et al., 2018). Both total RNA and RNC-associated RNA sequencing was conducted in three replicates on an Illumina Hiseq 2500 platform (Illumina, United States). The aim of total RNA sequencing was input for the calculation of translation efficiencies.

### m6A-seq

Approximately 0.4 g spikelets were pooled and used for RNA extraction. The integrity and quality of RNA was assessed using gel electrophoresis and a NanoDrop ND-1000 (Thermo Fisher Scientific, United States), respectively. High-quality RNA samples with OD260/OD280 ratio within the range of 1.8∼2.1 were used for library construction. RNA molecules were fragmented followed by a selection of fragments around 300 nt in size. Ribosomal RNA was removed from the selected RNA using Ribo-Zero rRNA Removal Kit for plant leaf (Illumina, United States) following manufacturer’s instructions. The retained RNA was equally divided into two samples, one of which was directly subjected to RNA-seq procedures, with the output used as the input in downstream analyses. To enrich the m6A methylated RNA fragments, RNA molecules in the second sample were incubated with m6A antibody provided by GenSeq m6A-MeRIP Kit (GenSeq™ Inc., China) for 2 h following the manufacturer’s instructions. The immunoprecipitated fragments were eluted and subjected to standard RNA-seq procedure.

### Small RNA-seq

Small RNA libraries were constructed following standard protocols. Briefly, total RNA extracted from ∼0.1 g spikelets was resolved on a 15% denaturing polyacrylamide gel, from which small RNA molecules (∼15–40 nt) were recovered and ligated into 3′ adaptors. The 3′ adaptor-ligated small RNA fragments were then reverse transcribed following the TruSeq Small RNA Library Prep Kit (Illumina, United States). The libraries were sequenced on an Illumina Hiseq 2500 platform with SE50 and the reads were trimmed to remove adaptors before downstream analyses. Three replicates were performed.

### Identification and analyses of m6A peaks

The PE150 reads were filtered using cutadapt (Martin, 2011) to remove adaptors and low-quality reads before they were aligned to the reference genome of hexaploid Chinese Spring wheat (IWGSC_REFSEQV1.0) (The International Wheat Genome Sequencing Consortium et al., 2018) using Hisat2. The package MACS (Model-based Analysis of ChIP-Seq) (Feng et al., 2011) was used for the identification of m6A peaks with the RNA-seq reads as background input. The peaks identified from two of the three plants were used for further analyses in this study.

The extent of m6A methylation of mRNA was calculated as described by Wan et al. (2015). Considering the lower coverage of m6A-seq reads, Wan et al. (2015) proposed a measurement of m6A methylation levels, Modified Fragment Per Kilobase of Transcript Per Million Fragments Mapped (MFPKM), which can be calculated by normalizing FPKM to the percentage of transcripts that are covered by m6A-seq reads. Therefore, we first calculated FPKMs of each transcript for both m6A-seq and RNA-seq reads using cufflinks (Trapnell et al., 2010) and the former was then normalized to MFPKM. The ratio of MFPKM of m6A-seq to FPKM of RNA-seq was used to measure the methylation extent of m6A-containing mRNAs.

The m6A methylation extent was also compared between homeologous sub-genomes. We used the triad list reported in Ramírez-González et al. (2018) and compared their m6A extent. The triads were categorized into seven groups as described by Ramírez-González et al. (2018) according to their m6A proportions. The categories used were; “A-dominant,” “B-dominant,” “D-dominant,” “A-suppressed,” “B-suppressed,” “D-suppressed,” and “Balanced” triads.

### Topological analyses of m6A sites

The consensus of m6A sites was identified following the method described by Luo et al. (2014). The 1000 most significant m6A peaks were selected for the search of motif ‘RRACH’ (R = A/G, H = A/C/U). The frequency was calculated for each of the motifs and visualized using weblogo (Crooks et al., 2004) with default parameters.

The m6A peaks were categorized into five groups according to their positions, 5′UTR, start codon, CDS, stop codon, and 3′UTR. The coordinates of these genomic elements were extracted from the genome annotation file and Bedtools^1^ was used to search for the overlaps between m6A peaks and each of the elements. To analyze the locations of m6As along mRNAs, the identified m6A peaks were used to represent the position of m6As, and the distance between them and start and stop codons were calculated.

### Small RNA quantification and target

The small RNA-seq reads were processed to trim the adaptor and only the reads that had been trimmed were kept for analyses. ShortStack (Axtell, 2013) was used for the quantification of miRNAs in each locus. The phasiRNA loci were identified using the methods described by De Paoli et al. (2009) and Li et al. (2016), which were implemented in a pipeline of pRNASeqTools obtained from github^2^ (Li et al., 2016). The periodicity of sRNA depth at phasiRNA loci was evaluated using the R package “mutltitaper” (Rahim, 2014). The potential targets of miRNA were predicted using the webserver of psTarget^3^ (Dai et al., 2018) with default parameters.

### Statistical analyses

Translation efficiency was measured as the ratio of FPKM of RNC-seq to FPKM of RNA-seq (Wang et al., 2013). Translation efficiencies between different sets of mRNAs, such as m6A methylated mRNAs, non-methylated mRNAs, and miRNA targeted mRNAs were statistically compared using Wilcoxon test (wilcox.test) implemented in R. The correlation between m6A extent and expression levels or translation efficiencies of transcripts were analyzed and visualized using R packages (cor.test).

### Diversity analyses

The synonymous (*Ks*) and non-synonymous (*Ka*) substitution rates were calculated using Kaks_calculator (Wang et al., 2010) after the alignment of homeologous genes in a triad. Two previous published datasets of the diversity of wheat exomes (He et al., 2019; Pont et al., 2019) were collected and used for the calculation of nucleotide diversity (π), fixation index-statistics (*Fst*), and Tajima’s *D* using vcftools (Danecek et al., 2011). The negative values of *Fst* were converted to zero before further analyses.

### Functional analyses

The gene functional annotations were obtained from the website of wheat genome^4^. Entries of InterPro IDs were included in the annotation document and translated into gene ontology entries and terms, which were used for GO enrichment analyses using a hypergeometrical test. Benjamini-Hochberg false discovery rate was applied to the enriched set at a threshold of *P* < 0.05. The protein sequences deduced from the m6A methylated mRNAs were queried against the KEGG (Kyoto Encyclopedia of Genes and Genomes) database to obtain their biological pathway identifiers. Enrichment of pathways was also performed using the hypergeometrical test package (phyper.test) implemented in R. The maps of pathways were downloaded from the KEGG website^5^.

### Data access

The raw sequence data was deposited in the National Center for Biotechnology Information (NCBI) Sequence Read Archive under the BioProject PRJNA642367.

## Results

### Transcriptome-wide identification of m6A methylation

**FIGURE 1:**
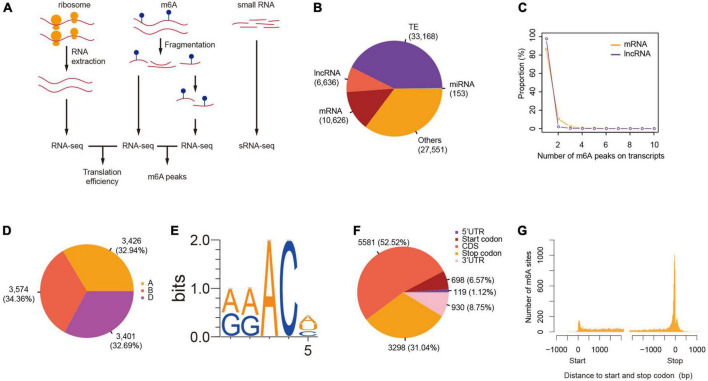
Transcriptome-wide identification of m6A in wheat. **(A)** A scheme showing the protocols of RNC-seq, RNA-seq, m6A-seq, and small RNA-seq. **(B)** The number of m6A peaks in different class of RNAs. **(C)** The number of m6As per transcript. **(D)** The distribution of m6A-containing mRNAs across sub-genomes. **(E)** The consensus motif of m6A sites. **(F)** The distribution of m6A peaks on mRNAs. **(G)** The distance between m6A peaks and start and stop codons.

We successfully constructed libraries for translatome quantification (RNC-seq), m6A-seq, as well as small RNA-seq in parallel (Figure 1A), and measured the levels of gene transcription and translation in order to investigate the roles of m6A in wheat gene translation. The m6A peaks were called after the mapping of both the RNA-seq and m6A-seq reads to reference genome of Chinese Spring wheat (RefSeqv1.0) (The International Wheat Genome Sequencing Consortium et al., 2018) and the peaks identified from two of the three plants were retained for further analyses (Supplementary Table 1). We identified 78,134 m6A peaks, among which 10,626 were found on 9,351 mRNAs. We also detected m6A peaks in other transcripts, for example, 6,636, 33,168, and 153 were found on transcripts from lncRNAs, transponsable elements, and miRNA precursors, respectively (Figure 1B). The numbers of m6A peaks on m6A methylated transcripts ranged from 1 to 10, although only a single peak was observed for more than 90% of the m6A methylated mRNAs (Figure 1C). This pattern was also observed in lncRNAs (Figure 1C). Globally, the m6A methylated transcripts were evenly distributed across all the 21 chromosomes and 3 sub-genomes (Figure 1D). “RRm6ACH” (R = A/G, H = A/C/U) has been identified as a consensus motif recognized by human methyltransferase-like 3 and 14 (Liu et al., 2014), and has also been reported in yeast (Schwartz et al., 2013) and in *Arabidopsis* (Luo et al., 2014; Wan et al., 2015; Wang et al., 2017). We also searched for this consensus in the top 1,000 significantly enriched m6A peaks. More than 90% of the m6A peaks had at least one of the “RRm6ACH” motifs with “GAACA” (35.8%) and “AAACA” (35.2%) being the most abundant (Figure 1E).

More than half of the m6A peaks were located at the coding sequence (52.52%), followed by the stop codon (31.04%), 3′UTR (8.75%), and the start codon (6.57%) (Figure 1F and Supplementary Table 2). Considering the difference in lengths of these regions, m6A peaks were enriched in the stop and start codons, as well as in the 3′UTR region (Figure 1G). The distribution pattern of m6A along transcripts was similar to that observed in *Arabidopsis* (Luo et al., 2014; Wan et al., 2015) but distinct from that found in mammals, such as mouse (Meyer et al., 2012) and human (Zhang et al., 2020), as well as yeast (Schwartz et al., 2013), suggesting conserved roles and mechanisms of m6A methylation in plants.

### Divergence of m6A methylation among homeologs

N6-methyladenosine-containing mRNAs were evenly distributed across all 21 chromosomes and three sub-genomes (Figure 1D). Variation of the expression of homeologous genes across sub-genomes had been reported in many polyploids including wheat (Ramírez-González et al., 2018). It was therefore of interest to explore the variation of m6A methylation across sub-genomes. Homeologous gene triads, which consist of homeologous genes from three sub-genomes, identified by Ramírez-González et al. (2018), provided ideal gene sets for this analysis due to similar sequences and functions of genes within each triad. In total, there are 18,474 triads reported by Ramírez-González et al. (2018), which consist of 55,422 genes that have 1:1:1 correspondence across the A, B, and D sub-genomes. Among them, 17,400 triads were located within syntenic blocks across the three sub-genomes while the other 1,074 triads were not. Our data indicate that there are 2,590 triads that had at least one gene containing m6A including 2,369 (91.47%) syntenic and 221 (8.53%) non-syntenic triads.

**FIGURE 2:**
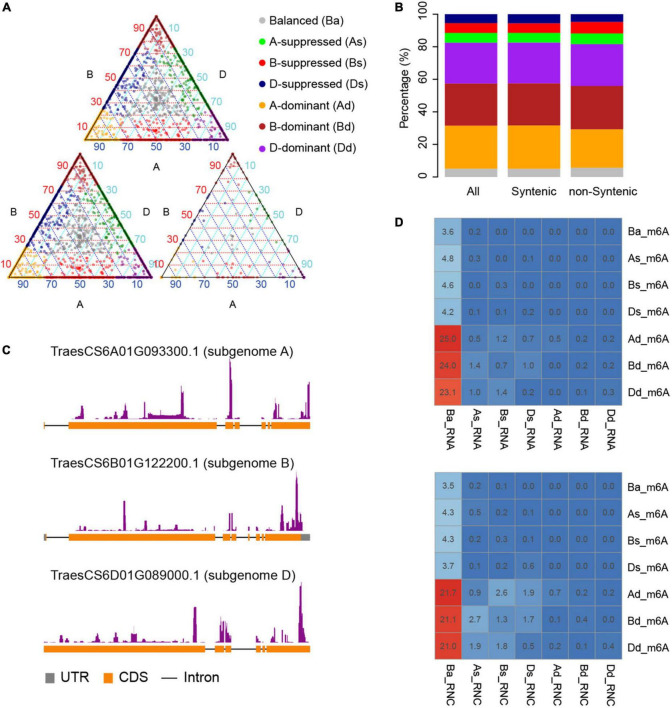
Divergence of m6A methylation across sub-genomes. **(A)** The classification of triads including m6A methylated mRNAs. Triads were categorized into seven classes (“A-dominant,” “B-dominant,” “D-dominant,” “A-suppressed,” “B-suppressed,” “D-suppressed,” and “Balanced”) according to the variance of m6A methylation among the homeologous genes. The triangle plot on top shows all the triads, and the bottom left and right triangle plot show the triads located within or outside of syntenic blocks, respectively. The points in the triangle plots indicate the relative degree of methylation across each gene triad (homeologous genes in three sub genomes). **(B)** The proportion of different classes in synthetic and non-synthetic triads. It uses the same color code as **(A)**. **(C)** One example triad showing the variance of m6A methylation positions. Homeologous genes TraesCS6A01G093300.1, TraesCS6B01G122200.1, and TraesCS6D01G089000.1 locate at sub-genome A, B, and D, respectively. **(D)** The proportion of overlaps between triads classified according to m6A methylation degree, transcription, or translation levels. Triads classified according to m6A degree, transcription (RNA), and translation levels (RNC) were labeled with “_m6A,” “_RNA,” and “_RNC,” respectively, and “Ba,” “As,” “Bs,” “Ds,” “Ad,” “Bd,” and “Dd” are short for “Balance,” “A-suppressed,” “B-suppressed,” “D-suppressed,” “A-dominant,” “B-dominant,” and “D-dominant,” respectively. The numbers in the cells show the percentage of triads for each class.

We further compared the degree of m6A methylation among the genes in each triad and found unbalanced methylation between homeologous genes, contrary to the even distribution of m6As across the three sub-genomes. We therefore classified the triads into seven groups (balanced, A-suppressed, B-suppressed, D-suppressed, A-dominant, B-dominant, and D-dominant) according to the degree of m6A methylation of genes in each triad using the criteria proposed by Ramírez-González et al. (2018). As a result, 98, 132, 126, 119, 731, 709, and 675 triads were classified into balanced, A-suppressed, B-suppressed, D-suppressed, A-dominant, B-dominant, and D-dominant groups, respectively (Figure 2A and Table 1). More than 75% of the triads fell into A-, B-, or D-dominant groups, suggesting one of the homeologous genes in these triads was methylated to an extent much higher than that of the other two genes. On the other hand, very few (∼5%) triads were categorized as “Balanced.” The variation of m6A methylation between sub-genomes appeared to be independent of the genomic locations of homeologous genes as reflected by the triads located within or outside of syntenic blocks (Figure 2B). To uncover whether the homeologous genes had similar patterns of methylation, we inspected in detail the locations of m6A sites of genes in 250 balanced triads. Although the methylation degrees were close across the homeologous genes, their locations varied substantially (Figure 2C). These results suggest substantial variation of m6A methylation between homeologous genes.

**TABLE 1:** Number of m6A-containing triads.

Group	Number of m6A-containing triads	Syntenic	Non-syntenic
Balanced	98	91	7
A-suppressed	132	115	17
B-suppressed	126	114	12
D-suppressed	119	111	8
A-dominant	931	869	62
B-dominant	709	655	54
D-dominant	675	614	61
Total	2590	2369	221

To explore the relationship between the diversification of m6A methylation and that of gene expression and translation levels, we also classified the genes in triads according to their transcription and translation levels (Ba_RNA, As_RNA, Bs_RNA, Ds_RNA, Ad_RNA, Bd_RNA, and Dd_RNA for transcription and Ba_RNC, As_RNC, Bs_RNC, Ds_RNC, Ad_RNC, Bd_RNC, and Dd_RNC for translation). It is interesting that most of the m6A methylated mRNAs were from Ba_RNA (Figure 2D), in which the three homeologous genes are conserved in their expression levels. Similarly, m6A sites were also enriched in mRNAs from Ba_RNC (Figure 2D) but with more methylated mRNAs from As_RNC, Bs_RNC, and Ds_RNC. These findings indicate that the dominant m6A methylation on one of the homeologous genes was related to its suppression of translation (Figure 2D), implying its role in suppressing the translation of mRNAs.

### Conservation of m6A methylated genes

**FIGURE 3:**
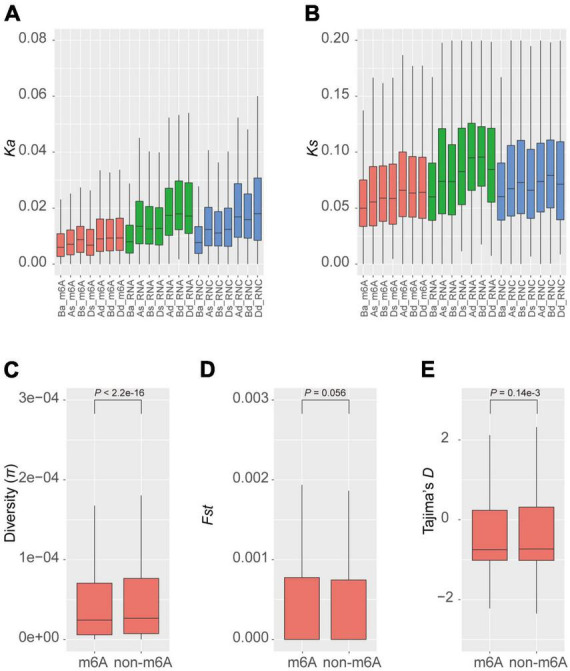
Diversity and selection of m6A methylated genes. Non-synonymous **(A)** and synonymous **(B)** substitution rates of homeologous genes in different groups of triads. The diversity **(C)**, fixation index statistics **(D)**, and Tajima’s *D* **(E)** of m6A methylated and non-methylated genes in the natural population.

Given that m6A methylation varies between homeologous genes, we further examined whether the methylated genes have selective advantages in the population. We calculated the synonymous (*Ks*) and non-synonymous (*Ka*) mutation rates between homeologous in different groups of triads. We found that generally the balanced groups (Ba_m6A, Ba_RNA, and Ba_RNC) had lower rates of both synonymous and non-synonymous substitutions (Figures 3A,B) and the dominant groups (Ad_m6A, Bd_m6A, Dd_m6A, Ad_RNA, Bd_RNA, Dd_RNA, Ad_RNC, Bd_RNC, and Dd_RNC) accumulated more mutations. We noticed that the substitution rates (both *Ka* and *Ks*) of m6A methylated triads were lower than those of transcribed and translated triads (Figures 3A,B). These results suggest a greater conservation of m6A methylated triads, particularly the triads with similar extent of methylation.

The conservation between homeologous genes in the balanced m6A methylated triads (Ba_m6A) raises the question of whether the m6A methylated genes are also more conserved than the non-methylated ones. To investigate this, we measured the diversity (π) of genes in a global collection of wheat (He et al., 2019; Pont et al., 2019). The average diversity of m6A methylated genes is 1.36e-4, which is significantly lower than that of the non-methylated genes (π = 1.51e-4, *P* < 2.2e-16, Wilcoxon test) (Figure 3C). To further explore whether these genes were preferentially selected during the process of wheat breeding, we calculated *Fst*, a statistic describing the genetic diversity partition within and among populations (Wright, 1951), for m6A methylated and non-methylated genes in the population (Figure 3D). The m6A methylated and non-methylated genes have average *Fst* values of 1.16e-2 and 1.13e-2, respectively, with no significant difference observed (*P* = 0.056, Wilcoxon test). On the contrary, the statistics of Tajima’s *D*, a statistic of neutrality test detecting genes that have not evolved neutrally in the population (Tajima, 1989) showed that the m6A methylated genes (Tajima’s *D* = −0.198) have been subjected to stronger purification than non-methylated genes (Tajima’s *D* = −0.151) (*P* = 0.14e-3) (Figure 3E). These results suggest that the m6A methylated genes might be widely engaged in conserved pathways or critical biological processes.

### Biological processes involving m6A-methylated genes

**FIGURE 4:**
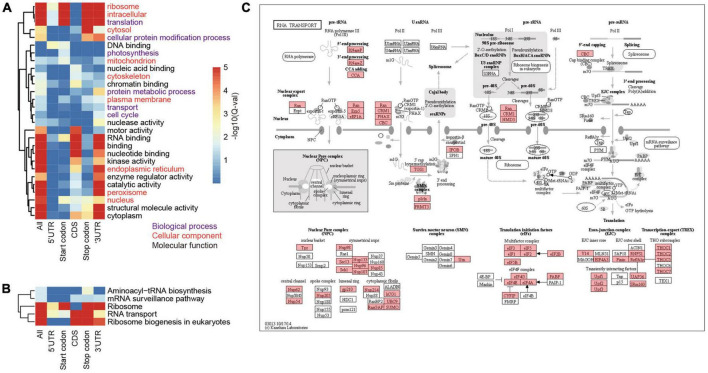
Functional enrichment of proteins encoded by m6A-containing mRNAs. **(A)** GO terms enriched by proteins encoded by different groups of m6A-containing mRNAs. **(B)** The enriched pathways related to translation. **(C)** The KEGG map of “RNA transport,” in which the m6A-containing genes are shaded with red.

To investigate the biological processes and pathways involving the m6A methylated genes, we annotated them according to the terms in GO database. As a result, we annotated 4,399 m6A-containing genes with known GO terms that were enriched in biological processes of translation, protein folding, and transport (Figure 4A and Supplementary Table 3) and their cellular locations were enriched in “ribosome,” “cytoplasm,” and “intracellular compartment” (Figure 4A). These results suggest a strong correlation between m6A methylation and protein synthesis, transport, and metabolism. Due to the substantial variations in the locations of m6A sites along the transcripts, we categorized the m6A-containing genes into genes with m6A at (I) 5′UTR, (II) start codon, (III) coding sequence, (IV) stop codon, and (V) 3′UTR, and analyzed their enriched functions separately. To obtain a more general view of the functions of these genes, we used only the slim terms in GO database for this analysis. We found that all the groups except group III (genes with m6A sites at coding sequences) were preferentially enriched in processes related to translation (Figure 4A and Supplementary Table 3). The distribution of m6A sites at gene CDS was abundant (Figures 1F,G) with diversified functions, but there was no biological process overrepresented by this group. Furthermore, genes with m6A sites at their stop codons and 3′UTRs were also enriched in “cellular protein modification process” (Figure 4A and Supplementary Table 3); group II and IV m6A-containing genes were also enriched in functions of “photosynthesis” and “cell cycle,” respectively. In line with the roles in translation of these m6A-containing genes, they were preferentially located in the ribosome, cytoplasm, and endoplasmic reticulum, where mRNA translation occurs (Figure 4A and Supplementary Table 3).

To further inspect the pathways that m6A-containing genes are involved in, we queried their sequences against the KEGG database (Figure 4C). The pathways related to translation were also over-represented by these genes. A total of 354 ribosome protein encoding genes were methylated (Supplementary Table 4). Genes involved in ribosome biogenesis, such as “RNA cytidine acetyltransferase,” “tRNA pseudouridine synthase B,” and “Ribosomal RNA-processing protein 7 A,” and RNA transport (Figure 4C), such as “Nuclear pore complex protein NUP96,” “Exportin-1” and “THO complex subunit 2-like protein,” as well as those involved in translation, including initiation and elongation factors, were also methylated (Supplementary Table 4; Figure 4B; and Supplementary Figure 1). Pathways of “carbon fixation” and “cell cycle” were significantly over-represented but by only a subset of m6A-containing genes. “Carbon fixation” pathway was over-represented by group II (Start codon) and “cell cycle” was over-represented by group III (CDS) (Supplementary Table 5). Taken together, our data show preferential methylation of genes in several pathways including translation and photosynthesis.

### Correlation between m6A methylation extent and gene transcription and translation levels

**FIGURE 5:**
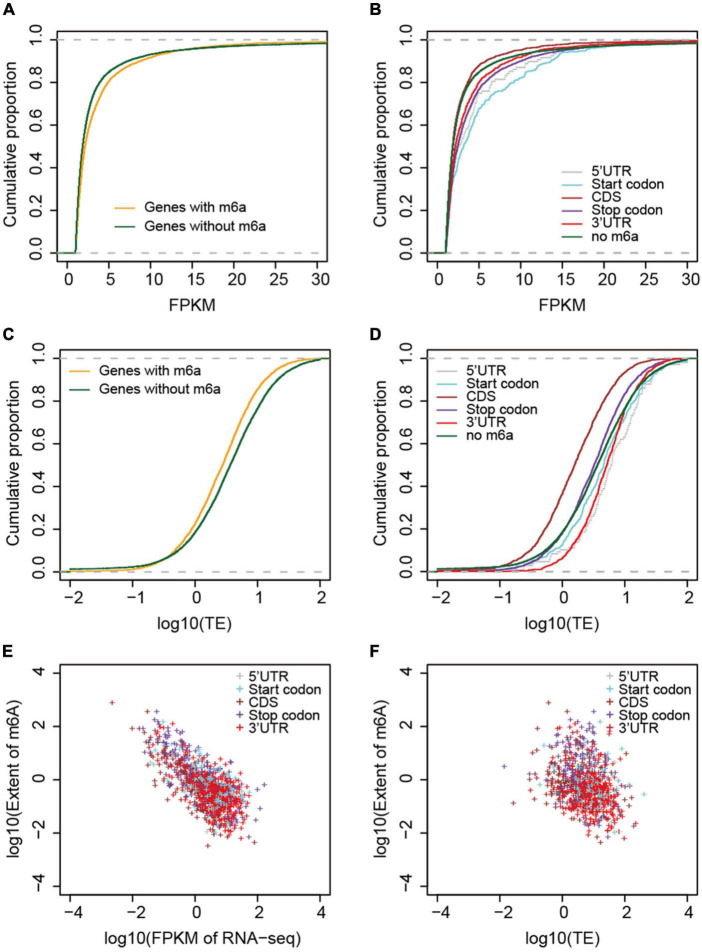
Correlation between m6A methylation and gene expression and translation levels. **(A)** Comparison of mRNA abundances (measured by FPKM) between non-methylated and m6A-containing mRNAs. **(B)** Comparison between different groups of m6A-containing mRNAs. **(C)** Comparison of mRNA translation efficiencies between m6A-containing and non-methylated mRNAs. **(D)** Comparison of translation efficiencies between different groups of m6A-containing mRNAs. **(E)** Correlation between the extent of m6A methylation and mRNA abundances, and **(F)** mRNA translation efficiencies. The different groups of m6A-containing mRNAs were color coded.

To examine the correlation between m6A methylation, transcription, and translation, we compared the expression and translation levels of mRNAs with and without m6A sites. Generally, the mRNAs with m6A sites had higher levels (mean FPKM = 6.17) of transcripts than those without (Figure 5A) (mean FPKM = 4.04) (*P* < 2.2e-16, Wilcoxon test, Table 2). This could be explained by the limitation of identification of m6A sites on low level mRNAs. However, the m6A-containing mRNAs belonging to group III (CDS) (mean FPKM = 3.13, *P* = 0.218, Wilcoxon test) did not show significant differences with non-methylated mRNAs in transcript abundance (Figure 5B and Table 2). The comparison between m6A methylation and translation, which was based on translation efficiency (FPKM of RNA/FPKM of RNC), revealed significantly lower translation efficiency for m6A methylated mRNAs (Figure 5C and Table 2) that was predominantly caused by the low translation efficiency of mRNAs with m6A sites on CDS or stop codon. The other groups of m6A-containing mRNAs showed comparable or higher translation efficiencies (Figure 5D).

**TABLE 2:** Transcriptomic and translatomic comparison between different groups of m6A-containing genes and non-methylated genes.

	Abundance difference^a^		Correlation			
	Transcriptome	Translation efficiency	Transcriptome		Translatome	
	*P-val*	*P-val*	*R*	*P-val*	*R*	*P-val*
5′UTR	4.00*e*−05	9.13*e*−05	–0.85135	0.1487	0.1001353	0.8999
Start codon	< 2.2*e*−16	0.01127	–0.59723	9.85*e*−09	–0.2276666	0.08289
CDS	0.218	< 2.2*e*−16	–0.74869	< 2.2*e*−16	–0.2164371	0.00025
Stop codon	< 2.2*e*−16	1.31*e*−06	–0.77995	< 2.2*e*−16	–0.2440065	0.000996
3′UTR	4.94*e*−09	5.54*e*−14	–0.62383	< 2.2*e*−16	–0.2755526	1.54*e*−06
All	< 2.2*e*−16	< 2.2*e*−16	–0.7109	< 2.2*e*−16	–0.2647965	1.32*e*−14

^a^Wilcoxon test.

We examined whether the m6A methylation extent was also correlated with the expression and translation levels of mRNAs. We therefore calculated the extent of m6A methylation extent by normalizing the level of m6A-seq to RNA-seq as described by Wan et al. (2015) in a study of the *Arabidopsis* m6A methylatome and compared it with the abundance of mRNAs and their translation efficiencies. mRNAs without m6A sites were excluded from this comparison. Although only a limited difference was observed for m6A methylated and non-methylated mRNAs in transcription level, we observed very strong and significant negative correlation between the m6A methylation extent and transcription level (Figure 5E). Specifically, for all groups, except group I (5′UTR) of m6A-containing genes, the extent of m6A methylation was negatively correlated with expression level (Figure 5E and Table 2). Group I also showed negative correlation but at a *P*-value > 0.01, which was probably due to the limited numbers of genes in this category. When we compared the m6A methylation extent and the translation efficiency of mRNAs, we observed significant negative correlations between m6A methylation extent and translation efficiency for groups III, IV, and V m6A-containing mRNAs (Figure 5F and Table 2), consistent with the earlier observations that mRNAs methylated at CDS and stop codon had lower translation efficiency. These correlations, however, were weaker than those observed between m6A methylation and transcription (Table 2). Overall, we conclude that m6A methylation is negatively correlated to gene transcription and can suppress the translation of mRNAs.

It was surprising that there were several RNC-seq reads mapped to lncRNAs, which suggested the active translation of lncRNAs, although their translation efficiencies were significantly lower than that of mRNAs (Supplementary Figure 2). We also identified m6A peaks on lncRNA transcripts (Figure 1G). To uncover the impact of m6As on the translation of lncRNAs, we compared the translation efficiencies between m6A-containing and non-methylated lncRNAs. However, we could not draw any conclusion due to the absence of m6A sites among the observed translated lncRNAs.

### Small RNA is a stronger inhibitor of translation than m6A methylation

The binding of small RNA is another important factor that suppresses the translation of mRNAs. To explore whether there is synergy between small RNA binding and m6A methylation in translation inhibition, we also sequenced the small RNA libraries in parallel. The size of small RNA in the spikelet peaked at 24 nt (Supplementary Figure 3), suggesting abundant phasiRNAs (Phased small-interfering RNAs) in this tissue. We first calculated the abundance of miRNAs by mapping them onto miRNA loci and found 4,775 miRNAs that were expressed in this tissue. As the materials used in this study were collected from spikes at flowering time, we also identified phasiRNAs, which is known to be abundant in gametes and play crucial roles in the regulation of mRNA translation (Li et al., 2016). Among them are the 21-nt phasiRNAs that are abundant in pistils during meiosis and in mature pollen (Zhai et al., 2015), but not during gametophytic development. In total, 13,961 phasiRNA loci including 959 21-nt phasiRNAs loci and 13,002 24-nt phasiRNAs were identified (Supplementary Table 6 and Supplementary Figure 4).

**FIGURE 6:**
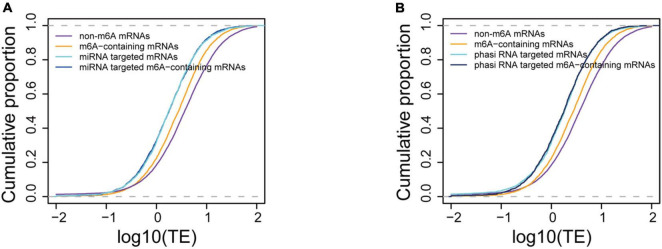
Comparison of translation suppression effects between m6A methylation and small RNA binding. **(A)** Comparison of translation efficiencies between m6A-containing, miRNA targeted, and m6A-containing mRNAs targeted by miRNA. **(B)** Comparison of translation efficiencies between m6A-containing and phasiRNA targeted mRNAs.

We further compared the translation efficiencies between the small RNA targeted and m6A methylated mRNAs. Our data showed that the translation efficiencies of miRNA targeted genes were significantly lower (*P* < 1e-16) than those of m6A methylated genes and other genes (Figure 6A), suggesting a stronger translation inhibition effect by miRNA. To investigate the synergism between these two translation inhibition machineries, we further compared the translation efficiencies between m6A methylated mRNAs that were also targeted by miRNAs and the miRNA targeted mRNAs. We observed no difference in translation efficiency between these two sets of mRNAs (4.07 for miRNA targeted mRNAs and 3.83 for m6A methylated miRNA target mRNAs, *P* = 0.7813, Wilcoxon test). It has been reported that m6A methylation usually occurs at the sites of miRNA targets in animals (Chen et al., 2015) and tend to change the structure from paired to unpaired RNA (Spitale et al., 2015), which might be beneficial for the binding of miRNAs. We therefore wanted to understand whether m6A methylation at miRNA targeting sites could enhance the binding of miRNAs, leading to stronger inhibition of translation. To test this hypothesis, we selected the m6A-containing mRNAs with miRNA targets located within the m6A peaks and compared them with other miRNA targeted mRNAs, and m6A methylated miRNA target mRNAs whose binding sites were not within the observed m6A peak. We observed no difference in the mRNA translation efficiency in these groups of mRNAs (3.83 for m6A methylated miRNA target mRNAs and 4.14 for mRNAs with miRNA binding sites overlapped with m6A peaks, *P* = 0.688, Wilcoxon test) (Supplementary Figure 5). The translation efficiencies of phasiRNAs targeted mRNAs were also compared with the m6A methylated mRNAs. We selected the top 300 abundant phasiRNAs and searched for their potential targets on mRNAs resulting in 33,421 potential targeting sites on 23,267 mRNAs for the selected phasiRNAs. It was surprising to find that although most of the phasiRNA loci identified in the current study were 24-nt loci, there were more 21-nt (173) than 24-nt phasiRNAs in the selected phasiRNAs. This suggests that the 21-nt phasiRNAs had higher expression levels than 24-nt phasiRNAs under the tested conditions. Similarly, we found that mRNAs potentially targeted by phasiRNAs had lower translation efficiencies (4.01) than the non-targeted mRNAs (9.41, *P* < 1e-16, Wilcoxon test). The effects in mRNA translation inhibition of phasiRNAs are comparable to that of miRNAs. Like the mRNAs targeted by miRNAs, the translation efficiencies of phasiRNA-targeted mRNAs (3.6) were also lower than m6A-containing mRNAs (5.89) and no synergism was found either (Figure 6B). Overall, our results suggested that small RNAs, including both miRNA and phasiRNA, are stronger inhibitors of mRNA translation than m6A methylation and that there was no synergism between these two machineries.

## Discussion

N6-methyladenosine methylation is the most prevalent and reversible internal modification of mRNAs of eukaryotes and plays important roles in post-transcription regulation (Gilbert et al., 2016). The components in m6A methylation machinery are complete in plants and are conserved across eukaryotic lineages (Yue et al., 2019), but appear to be distinctly different in their functions in animals and plants due to their distinct biological characters, such as photosynthesis in plants.

### Distinct topological pattern of m6A localization in plants

The motif “RRACH” is known to be the consensus of m6A sites (Gilbert et al., 2016). This consensus was also found in more than 90% of the m6A sites in wheat mRNAs and has also been reported in many other studies in plants (Luo et al., 2014; Wan et al., 2015; Murik et al., 2020). Despite the conservation of this consensus across animals and plants, the preference for degenerate nucleotides varies. In plants, the nucleotides immediate upstream of m6A favor adenine against guanine (Figure 1E) (Luo et al., 2014; Wan et al., 2015; Murik et al., 2020) while animals and yeast prefer more guanines at these sites (Schwartz et al., 2013; Liu et al., 2014). Furthermore, the recent identification of different consensus motifs from m6A sites in maize transcriptome (Du et al., 2020; Murik et al., 2020) implies the existence of different machineries.

N6-methyladenosine is preferentially located at the 3′UTR region near the stop codons in mammals (Meyer et al., 2012) and yeast (Schwartz et al., 2013). Bodi et al. (2012) reported a bias of m6A distribution toward the 3′ end of mRNA in *Arabidopsis* by separately measuring the levels of m6As in 5′ end, middle, and 3′ end mRNA fragments. The application of high-throughput sequencing techniques recently enabled transcriptome-wide identification of m6A sites on mRNAs and revealed a distinct distribution pattern in plant mRNAs. In addition to the enrichment around the stop codon and 3′UTR as was found in animals and yeast, m6A sites were also enriched in the start codons. Our data has also shown significant enrichment of m6As at start codons (Figure 1G). This pattern is most likely conserved across plant lineages because it has also been reported in both dicots (*Arabidopsis*) (Luo et al., 2014; Wan et al., 2015) and monocots (rice, maize, and wheat) (Li et al., 2014; Luo et al., 2020; Figure 1G).

### m6A methylation varied across sub-genomes but was conserved in the population

A previous comparison between two *Arabidopsis* ecotypes, Can-0 and Hen-16, reported a conserved pattern of m6A methylation between them (Luo et al., 2014), although variations in m6A methylation were found in different lines of maize (Luo et al., 2020). The conservation among homeologs in different sub-genomes has not been reported due to a lack of research in polyploids. In the current study, we compared homeologs in A, B, and D sub-genomes in terms of their methylation extent and locations. By comparing 7,338 genes belonging to 4,942 triads, only few balanced triads (all three genes methylated to comparable extents) were identified, suggesting very limited conservation across sub-genomes despite the even distribution of the number of m6A-containing genes (Figure 1D). We also observed variations in the m6A positions on mRNAs of genes in balanced triads (Figure 2B). On the other hand, most of m6A methylated genes were in the group of balanced triads of transcription and translation. The diverged levels of transcription and translation could be due to the diversified functions of homeologous genes and the genes in the balanced group were thought to be more conserved (Ramírez-González et al., 2018). As m6A methylation is related to the translation of mRNAs, the enrichment of m6A methylation in the balanced groups could play a role in regulating the translation, and thus lead to the diversification of the homeologous genes with balanced expression levels. In line with the critical roles played by the m6A methylated genes (Figure 4A), their diversity in the natural population is lower than the non-methylated genes; because of the engagement of several important biological processes, the m6A methylated genes were not preferentially selected by humans during the breeding practices.

### Impacts of m6A on mRNA translation

N6-methyladenosine methylation is known to play a role in regulating mRNA translation in mammals (Wang et al., 2015). The human YTH domain family protein 2 (YTHDF2) recognizes m6A-containing mRNAs and promotes the loading of ribosomes; it can also increase translation by interacting with initiation factors to facilitate translation initiation (Wang et al., 2015). Although m6As are preferentially located at the 3′UTR region near the stop codons, those at 5′UTR may also play profound roles in promoting translation. mRNAs with m6As within 5′UTR have been reported to have higher translation efficiency (Meyer et al., 2015) and can promote cap-independent translation initiation, particularly under stress conditions (Meyer et al., 2015). The presence of m6A in a mouse mRNA increased its translation efficiency in the rabbit reticulocyte translation system but failed to increase the translation efficiency in the wheat germ system (Heilman et al., 1996), which suggests differences in the translation regulation machinery in animals and plants, which should be further investigated. Our data also revealed a reverse correlation between m6A methylation and translation efficiency of wheat mRNAs in general (Figure 5F), which is inconsistent with a report from humans (Wang et al., 2015). However, when we divided the m6A-containing genes into subgroups and analyzed separately, different locations of m6A showed different impacts on translation (Figure 5D). The repression of mRNA translation by m6A methylation was predominantly as a result of m6A methylation in coding sequences and stop codons. N6-methyladenosine within codons could compromise the accuracy of translation and hampered tRNA accommodation and translation elongation (Choi et al., 2016). On the other hand, mRNAs with m6A within 5′UTR and start codons showed higher translation efficiencies than those not methylated, which is in line with reports from mammals (Meyer et al., 2015) and maize (Luo et al., 2020). Accordingly, among all these subgroups, mRNAs with m6A within 5′UTR had the highest translation efficiency. The similarities in the consequence of 5′UTR m6A methylation in the current study with those earlier reported in human cells imply a conserved regulatory machinery of promoting cap-independent translation, but this will require further investigation.

Both the translation and m6A methylation were found for lncRNAs in our study despite the lack of overlap between these two sets of lncRNAs. Although no meaningful conclusion could be made with the data presented here, it is reasonable to speculate that m6A methylation on lncRNAs may play similar roles in suppressing translation as observed in mRNAs. As the translation levels of lncRNAs were generally much lower than those of mRNAs, the suppression by m6A would further reduce their translation to undetectable levels. Further studies focusing on lncRNAs would provide more insights into the roles of m6A in lncRNA regulations.

### A machinery of secondary amplification of translation regulation by m6A

**FIGURE 7:**
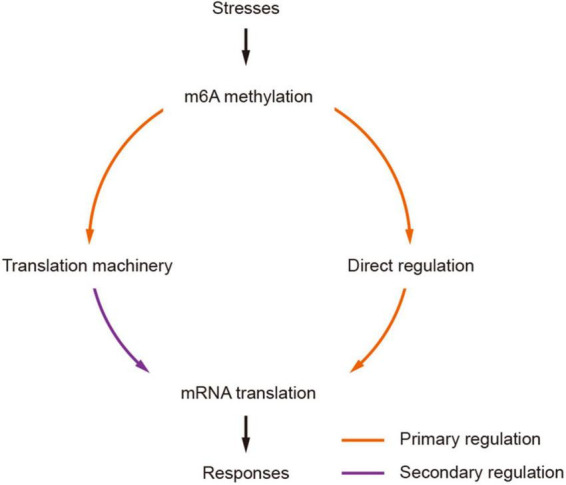
A model of secondarily amplified regulation of mRNA translation by m6A methylation. The changes of m6A methylation status (methylated/demethylated) induced by stresses are transferred to stronger translation regulators by primarily affecting the translation of mRNAs encoding components in translation machinery, which would result in more profound and strong impacts to mRNA translation, facilitating effective and prompt responses to stresses.

The impact of m6As on translation was also found in the modification of mRNAs encoding proteins involved in translation machineries. Wheat m6A-containing genes were generally over-represented by genes engaged in ribosome biogenesis, such as TraesCS7A01G309900 encoding a 60 S acidic ribosomal protein (Supplementary Table 4), and RNA export from the nucleus (Supplementary Table 4). It is interesting that all these subgroups except group III (CDS) were enriched in the processes of “translation” and “RNA transport.” As different groups have different impacts on translation, the universal distribution of m6A on mRNAs in the pathways of “translation” and “RNA transport” could provide a basis for rapid regulation of the translation of these mRNAs, which would possibly amplify the translational regulation of mRNAs. Taken together, our results suggest that m6A methylation can potentially regulate mRNA translation in two manners: (1) methylation of target mRNAs, which can enhance the separation of methylated mRNAs from translatable pools and the assembly of stress granules (Anders et al., 2018; Ries et al., 2019; Fu and Zhuang, 2020); (2) methylation of mRNAs encoding proteins in translation machineries. Indeed, recent studies of rice also indicated that the m6A methylation levels on mRNAs encoding proteins engaged in translation processes were significantly changed under either biotic (infection with rice stripe or rice black-streaked dwarf virus) (Zhang et al., 2021) or abiotic (cadmium stress) (Cheng et al., 2021) stress conditions. We propose a model of secondary amplification of translation regulation by the changes to m6A methylation status (Figure 7). In this model, the changes in m6A methylation status (methylated/demethylated) induced stresses would primarily lead to the alteration of translation levels of mRNAs, among which the increased/decreased accumulation of products involved in translation machinery could secondarily regulate mRNA translation by enhancing/reducing the general translation activities in the cell.

Overall, in this study, we presented a transcriptome-wide profiling of m6A and small RNA in hexaploid wheat and reported translation regulation as one of its major roles in wheat. m6A methylation can potentially regulate mRNA translation directly or indirectly through three different approaches. First, the presence of m6As can suppress or promote the translation of mRNAs depending on their locations; second, the mRNAs encoding proteins involved in translation machineries are primarily methylated, resulting in more profound and global impacts of mRNA translation. We proposed a secondary amplified mRNA translation regulation model triggered by the changes in m6A methylation status which allows rapid translatomic responses to stresses. Therefore, the components of RNA methylation pathways can potentially be used as candidates for future engineering for stress tolerance in plants.

## Data availability statement

The original contributions presented in this study are publicly available. The raw sequence data was deposited in the National Center for Biotechnology Information (NCBI) Sequence Read Archive under the BioProject PRJNA642367.

## Author contributions

PY, BS, and Y-CL conceived this study. TH and W-JH prepared the materials and conducted the experiments. TH, CL, and J-BZ analyzed the data. TH, PY, and BS wrote the manuscript. Y-CL revised the manuscript. All authors reviewed the manuscript.

## Footnotes

1
https://bedtools.readthedocs.io/en/latest/

2
https://github.com/grubbybio/pRNASeqTools

3
http://plantgrn.noble.org/psRNATarget/

4
https://urgi.versailles.inra.fr/download/iwgsc/IWGSC_RefSeq_Annotations/

5
https://www.kegg.jp/

## Supplementary material

Supplementary Figure 1 KEGG pathways overrepresented by m6A methylated mRNAs. **(A)** Ribosome biogenesis. **(B)** Ribosome components. **(C)** RNA degradation. The m6A-containing genes are shaded with red.

Supplementary Figure 2 Comparison of translation efficiencies between mRNA and lncRNAs.

Supplementary Figure 3 Size distribution of small RNAs.

Supplementary Figure 4 Two examples of PHAS loci. **(A)** The depth distribution of a 21-nt PHAS locus “chr1A_418997663_418997852_21.” **(B)** The depth distribution of 24-nt PHAS locus “chr7A_563367941_563368061_24.” The periodicity of small RNA on the loci of **(C)** “chr1A_418997663_418997852_21” and **(D)** “chr7A_563367941_563368061_24.” The red and blue dashed lines in **(C,D)** indicate the expected frequencies for 21-nt (1/21) and 24-nt (1/24) small RNAs. The peaks at these indicated positions suggest the small RNAs appear at these loci in an interval of 21 or 24 nt, consistent with their sizes.

Supplementary Figure 5 Comparison of translation efficiencies between m6A-containing mRNAs with miRNA binding sites within or outside of the m6A peaks.
