# Supplementary figures and images for: Transcriptome-wide analyses of RNA m6A methylation in hexaploid wheat reveal its roles in mRNA translation regulation
Source: Front Plant Sci. 2022 Aug 25;13:917335. doi: 10.3389/fpls.2022.917335 (PMC9453602; doi:10.3389/fpls.2022.917335)

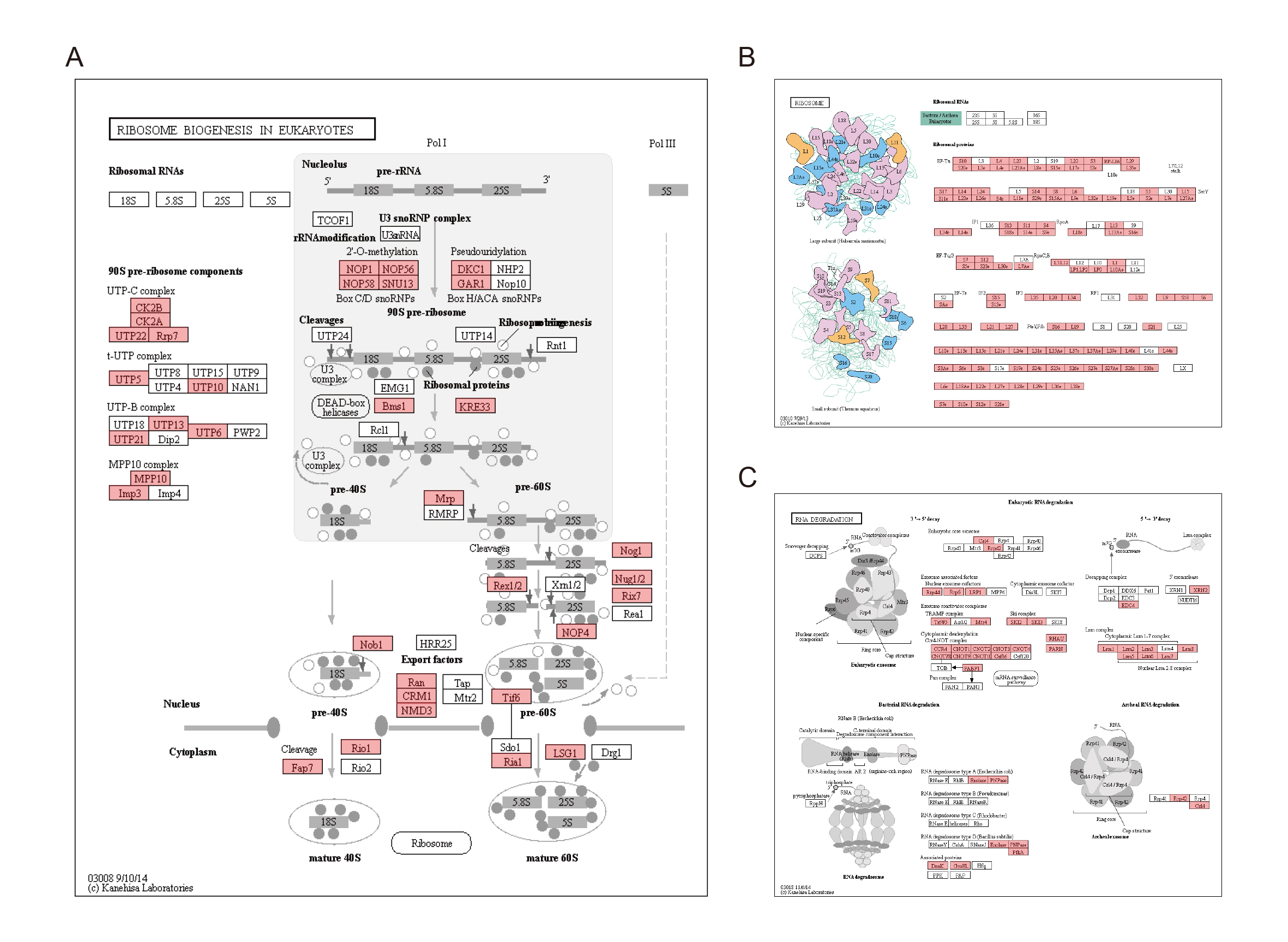

Supplement: Supplementary Figure 1 — KEGG pathways overrepresented by m6A methylated mRNAs. (A) Ribosome biogenesis. (B) Ribosome components. (C) RNA degradation. The m6A-containing genes are shaded with red. [file Image_1.JPEG]

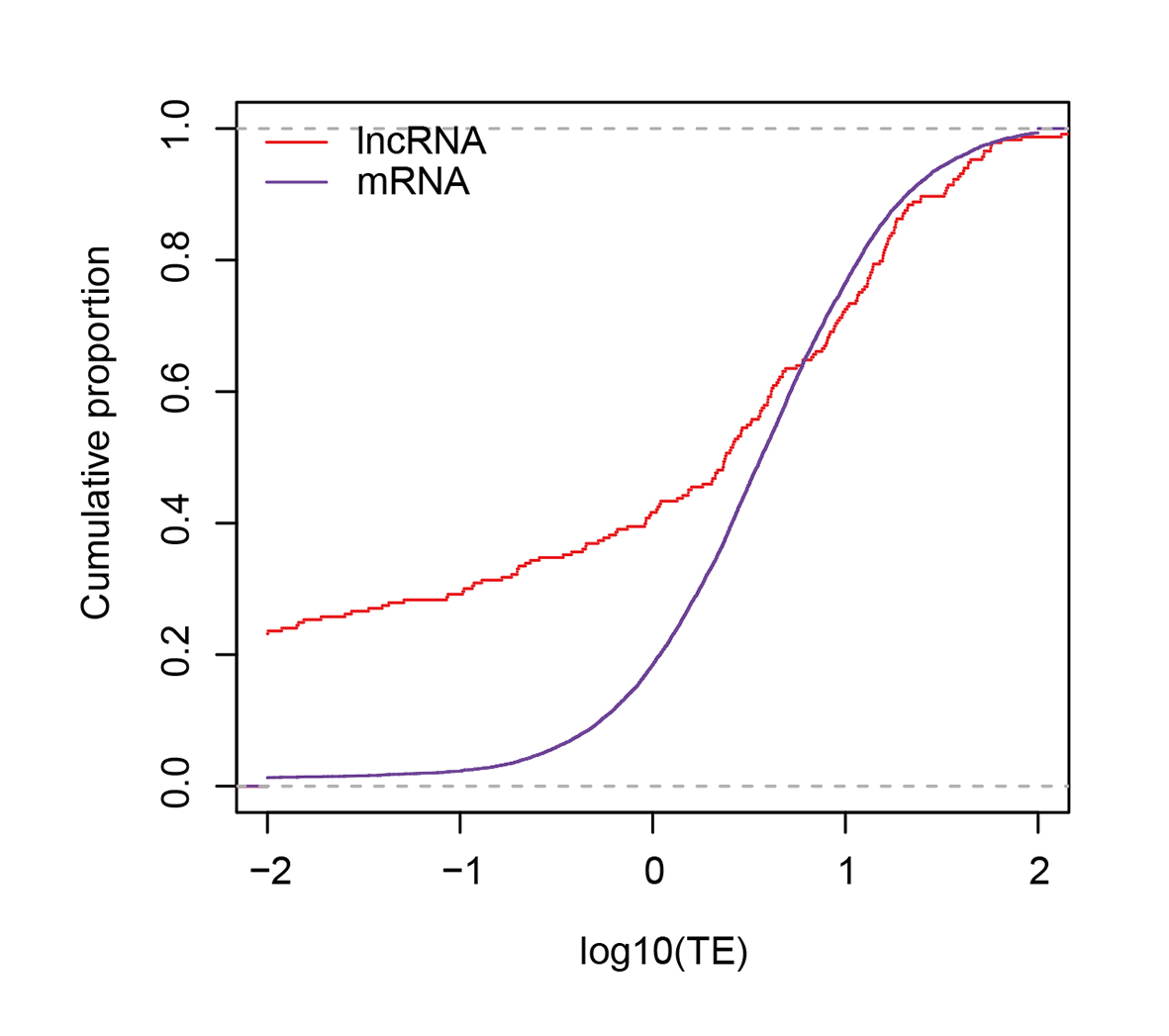

Supplement: Supplementary Figure 2 — Comparison of translation efficiencies between mRNA and lncRNAs. [file Image_2.JPEG]

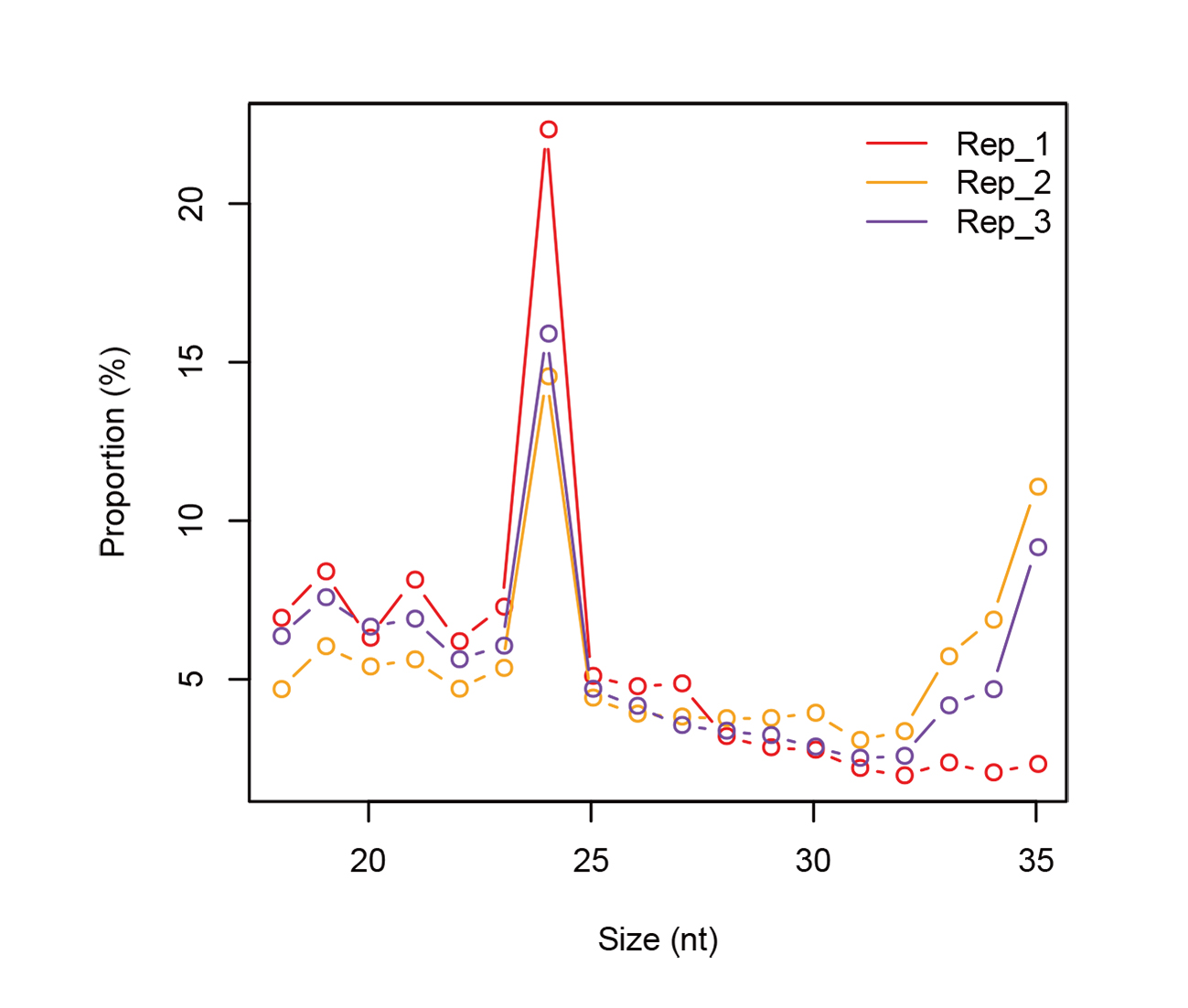

Supplement: Supplementary Figure 3 — Size distribution of small RNAs. [file Image_3.JPEG]

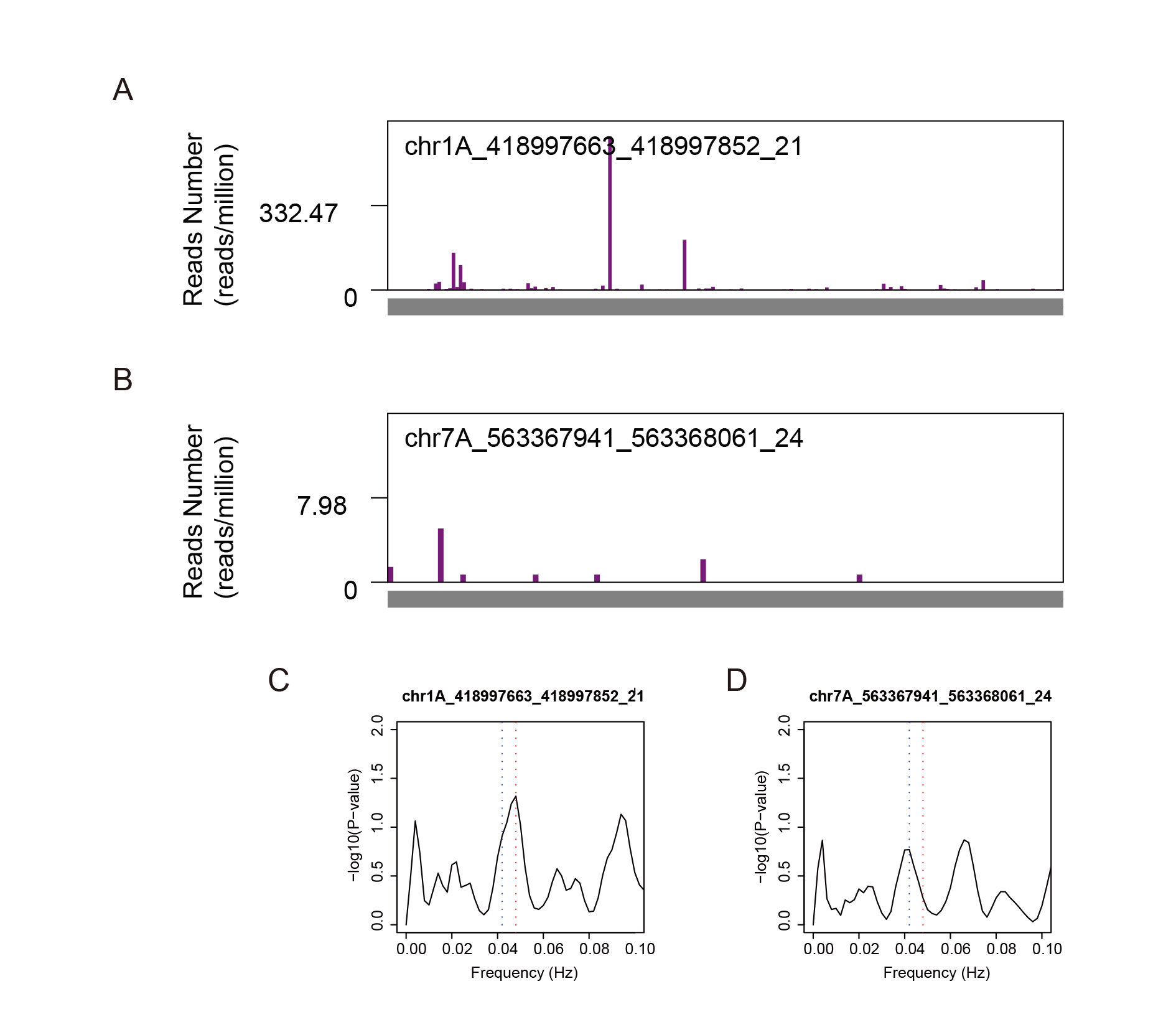

Supplement: Supplementary Figure 4 — Two examples of PHAS loci. (A) The depth distribution of a 21-nt PHAS locus “chr1A_418997663_418997852_21.” (B) The depth distribution of 24-nt PHAS locus “chr7A_563367941_563368061_24.” The periodicity of small RNA on the loci of (C) “chr1A_418997663_418997852_21” and (D) “chr7A_563367941_563368061_24.” The red and blue dashed lines in (C,D) indicate the expected frequencies for 21-nt (1/21) and 24-nt (1/24) small RNAs. The peaks at these indicated positions suggest the small RNAs appear at these loci in an interval of 21 or 24 nt, consistent with their sizes. [file Image_4.JPEG]

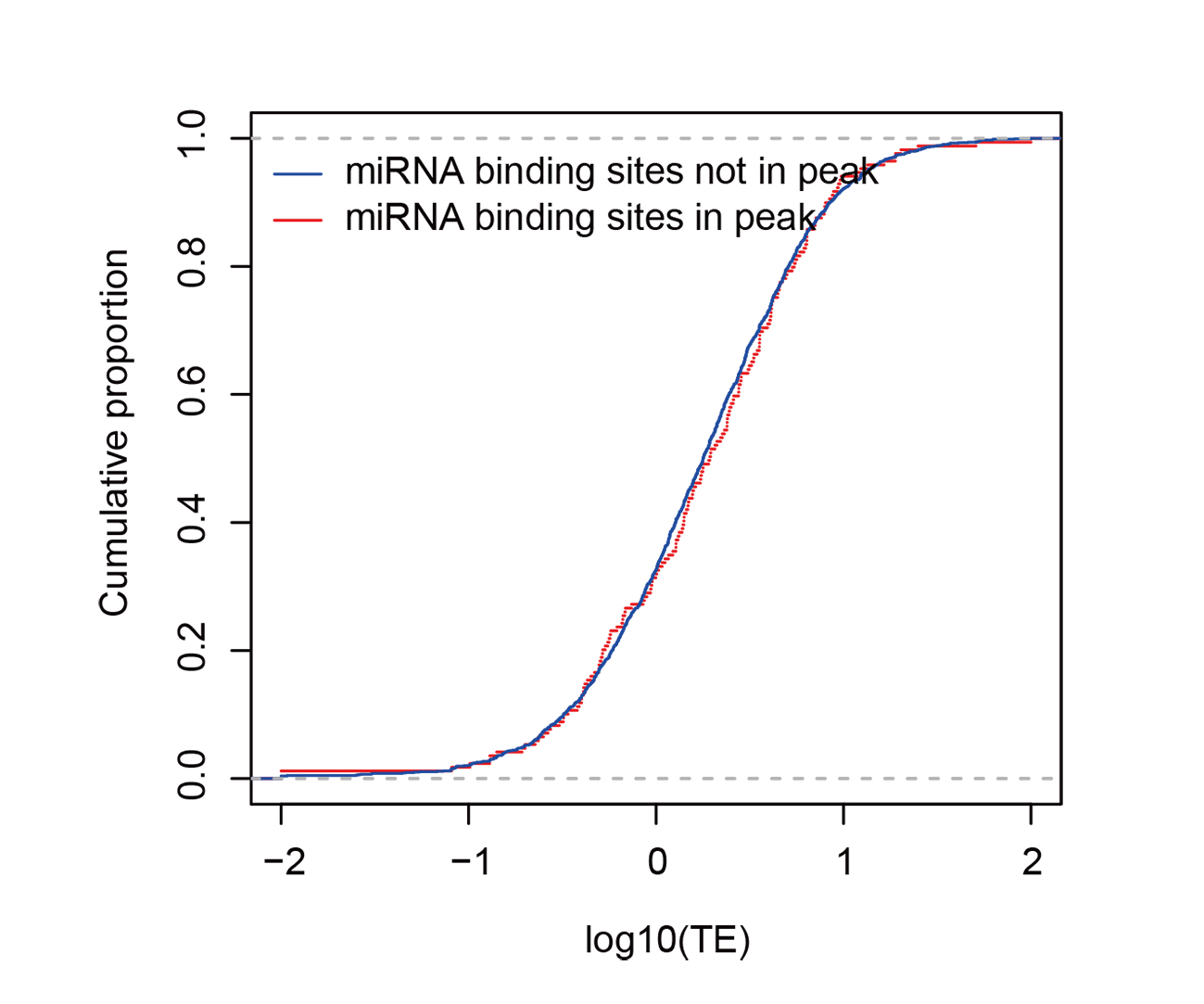

Supplement: Supplementary Figure 5 — Comparison of translation efficiencies between m6A-containing mRNAs with miRNA binding sites within or outside of the m6A peaks. [file Image_5.JPEG]
